# A randomised Study Within a Trial (SWAT) to determine if participant information leaflet design affects recruitment rate into an interventional trial taking place in a UK emergency department

**DOI:** 10.1186/s13063-025-09412-6

**Published:** 2026-02-06

**Authors:** Rachelle Sherman, Andrew Tabner, Apostolos Fakis, Adwoa Parker, Graham Johnson

**Affiliations:** 1 Derby Clinical Trials Support Unit, University Hospitals of Derby and Burton NHS Foundation Trust, Derby, UK; 2 Emergency Department, University Hospitals of Derby and Burton NHS Foundation Trust, Derby, UK; 3 York Trials Unit, Department of Health Sciences, University of York, York, UK

**Keywords:** Recruitment, SWAT, Consent, Participant information

## Abstract

**Background:**

Exploring barriers and enablers to participant recruitment into trials is a common discussion point in trial methodology. Participant information leaflets (PIL) can be long, have complexity above the average UK reading age, and may discourage engagement with research.

This Study Within a Trial (SWAT) explored whether changing the design of a PIL influences recruitment rate and its value in patient decision-making. It was conducted within a host trial taking place in an emergency setting, where time is at a premium, and decisions on trial participation are needed more quickly than in most non-emergency settings.

**Methods:**

We have conducted a randomised SWAT, comparing the standard format PIL with one that has been adapted to be visually appealing, with improved readability and reduced word count. Patients considered eligible for the host trial were provided with a randomly allocated PIL type; consent rates were compared. Those consenting to take part in the host trial were asked to complete a questionnaire to explore the value of the PIL in their decision-making to take part in the trial; responses were compared across the two information sheets. The sample size was dictated by host trial recruitment.

**Results:**

Between September 2019 and September 2022, with a brief pause during the COVID19 pandemic, 271 participants were randomised to receive either the optimised PIL (*n* = 138) or the conventional PIL (*n* = 133). The recruitment rates were 47.1% (65/138) in the optimised PIL group and 48.9% (65/133) in the conventional PIL group; this difference was not statistically significant (*p* = 0.771). There were no significant differences in responses from participants recruited to the host trial who completed the Decision-Making Questionnaire.

**Conclusion:**

Improving the readability and visual presentation of the participant information sheet provided to participants had no effect on recruitment rate, and did not appear to impact decision-making of recruited participants.

**Supplementary Information:**

The online version contains supplementary material available at 10.1186/s13063-025-09412-6.

## Background

Clinical trials are an essential component of evidence-based medicine; however, a significant proportion fail to recruit to time and target. A recent systematic review of trials published in the NIHR Journals Library between 1997 and 2020 indicated that although there was an improvement to recruitment in recent years, only 53% of trials reached their original sample size target, and a third required an extension to their recruitment period [1]. Poor recruitment into studies can lead to costly extensions and risks them being underpowered [2]. There are many barriers to study recruitment which may be at the study, site or patient level [3–5], and addressing recruitment challenges in clinical research has been recognised as one of the priorities for trial methodology research in the UK [6].

Participant information leaflets (PIL) given to potential participants are often long, complex and visually unappealing, and it is well reported that their reading age is higher than it should be [7]. Just under half of UK adults (43%) have literacy skills equivalent to that of an 11–14-year-old or lower GCSE level [8]. A well-designed, but simpler, information sheet and consent form may speed up the consent process without compromising patient understanding [9, 10]. It could even improve understanding [11]. A more efficient consent process could address barriers related to time and resource limitations for research [5]; this is particularly relevant in situations where study interventions are time critical, e.g. in the emergency setting.

The move towards shorter, more readable PILs has been recognised by the UK’s Health Research Authority (HRA) as they released the Participant Information Design and Review Principles in 2023 [12]. These were created to support researchers and Research Ethics Committees (RECs) and highlight the need to ensure information provided supports participants making informed choices about their involvement, and to encourage staff to engage in recruitment activities. The information provided to participants plays an important role as a point of reference for them [13], and researchers must therefore balance providing sufficient information with avoiding off-putting lengthy documentation.

To ensure that clinical research conducted is high quality, robust and cost-effective, it is important that methodology research, or “research on research” is undertaken to provide evidence to inform the decisions made by those designing them. The potential for modifying information sheets to improve recruitment to trials has been explored by Treweek et al. (2018), who report (with high certainty) that using a user-testing approach to develop the PIL made little or no difference to recruitment [14]. Similarly, using a brief PIL and a PIL developed with user feedback showed little to no impact on recruitment with moderate certainty [14]. However, the trials reviewed were conducted mainly in primary care, and none in an emergency department setting, which itself is an area as distinct as primary and secondary care, so we felt that further evaluation was necessary in this area. Similar findings, where no difference to the recruitment rate is seen when modifying consent forms, might suggest that participants do not value them highly in their decision-making process or read them fully [11].

We conducted a randomised Study Within a Trial (SWAT) within the SARC trial to compare the effect of improving readability and optimising the design of the PIL on recruitment and participant decision-making. The SARC trial was a single centre, randomised, placebo-controlled phase II trial assessing the efficacy of intravenous salbutamol as an analgesic adjunct for patients presenting to the emergency department with renal colic pain requiring intravenous analgesia [15].

## Methods

For our SWAT, participants were randomised to receive a PIL with an optimised design (the “optimised” PIL) or one based on the standard Health Research Authority (HRA) template (the “conventional” PIL). The SWAT protocol was embedded within the SARC protocol [16] and was approved alongside the SARC trial (IRAS 252075) by the West of Scotland Research Ethics Committee 1 (19/WS/0087). There were no amendments to the SWAT, which is registered as SWAT101 in the SWAT Repository [17].

### Participants

Patients identified as potentially eligible for the SARC trial were randomised to receive either the optimised PIL or the conventional PIL (Additional File 1). We did not inform participants that they were randomised to receive one of two participant information leaflets. All participants who consented to take part in SARC were asked to complete a Decision-Making Questionnaire (DMQ) at the end of their trial participation (Additional File 2). We opted not to approach those declining to take part in SARC to complete the DMQ, to reduce burden on site staff, as additional consent and data collection would have been required.

### Intervention

We designed the optimised PIL based on work by other researchers and with a view to improve readability by reducing the number of words per sentence, using familiar words and phrases, a columnar layout, and clear headings [11, 18–20]. Word count and readability statistics were determined using Microsoft Word’s in-built tool, chosen for ease and availability (Table 1). The optimised PIL was printed in full colour by a local printing company and presented as a folded A3 sheet.

**Table 1:** Comparison of the optimised PIL and the conventional PIL

	**Optimised PIL (A)**	**Conventional PIL (B)**
**Number of pages**	4	9
**Word count (exc. titles)**	1563	1952
**Number of sections**	8	15
**Sentences per paragraph**	1.3	2.1
**Format/structure/layout**	A3 folded booklet, printed in colour	A4 pages, double sided, printed black and white
	Left justified columnar layout	Left justified single column block text
	Bullet points	No bullet points
**Flesch Reading Ease**	62.1	58.8
**Flesch-Kincaid Grade Level**	9.8	10.4
**Passive sentences**	17.7%	38.2%

### Comparator

The conventional PIL was designed independently from the SWAT trial team, using the HRA standard template available at the time. It was printed in black and white on double-sided A4 paper, reflecting usual practice.

Both PILs were reviewed by the SARC PPI group and improvements were made based on their suggestions.

### Comparison of the patient information leaflets

The optimised PIL had an improved readability score, reduced word count, shorter paragraphs and fewer pages compared to the conventional PIL (Table 1). The Flesch-Kincaid grade level was slightly lower in the optimised PIL (9.8 vs 10.4), indicating that it is suitable for a lower grade level of education [21]. Whilst the Flesch Reading Ease score is also higher (and therefore better) in the optimised PIL, this is a modest improvement, and both sit close to the borderline of "standard" and "fairly difficult" categories [22].

### Outcomes

The primary outcome was the proportion of patients who agreed to take part in the SARC trial. Secondary outcomes were responses on the Decision-Making Questionnaire (DMQ), provided as Additional File 2. The DMQ was developed from a questionnaire used in the “TRECA” study [23] designed to assess the impact or value of the PIL in the decision-making of the patient. It was provided at the same timepoint as other SARC questionnaires at the end of trial participation.

### Sample size

The SWAT sample size was dependent on the SARC trial; therefore, no formal sample size calculation was performed, in line with accepted SWAT methodology [24, 25]. The sample size for the secondary measure, the DMQ, was limited to those who consented to take part in the SARC trial and agreed to complete the questionnaire.

### Randomisation

We used the web-based platform, Sealed Envelope™, to generate the randomisation list, using a randomly varying size block randomisation (2, 4, 6, or 8) allocated on a 1:1 basis. The Trial Manager (RS) used the sequence to sort the PILs into opaque envelopes independent of the recruiting team. The envelopes were labelled with sequential identification numbers (SWAT ID), and recruiters were instructed to provide the envelopes in consecutive order based on the SWAT ID. Upon identifying potential participants for the SARC trial, the recruiters took the next available envelope and used the PIL contained within to provide trial information to the patients.

### Analysis

Analysis was conducted on an intention-to-treat basis, including all randomised participants according to the PIL groups to which they were randomised. A pre-planned interim analysis of the primary outcome was performed after 6 months of recruitment. The purpose of this analysis was to assess whether there was a statistically significant difference in recruitment rates between the two participant information leaflets (PILs), which could potentially influence the conduct of the host trial. Specifically, the interim analysis served as a safeguard to identify any adverse impact of the optimised PIL on recruitment, ensuring that the integrity of the host trial remained unaffected by the SWAT intervention.

For analysis of the primary outcome, a chi-squared test and logistic regression, adjusted by age, were used to compare recruitment rates between groups; results were presented as odds ratios together with their 95% confidence intervals and *p*-values.

For the secondary outcomes, the observed frequencies of answers to each question in the DMQ between the two groups were compared descriptively; Fisher’s exact and Kruskal-Wallis tests were used at the 5% significance level.

We used the CONSORT checklist when writing our report [26] alongside the SWAT reporting guidelines [27].

## Results

### Recruitment

Recruitment to the SWAT started in September 2019 in line with recruitment to SARC, paused in March 2020 due to the COVID-19 pandemic, then recommenced in November 2020. SWAT recruitment stopped in April 2022.

### Participant flow

**Fig. 1:**
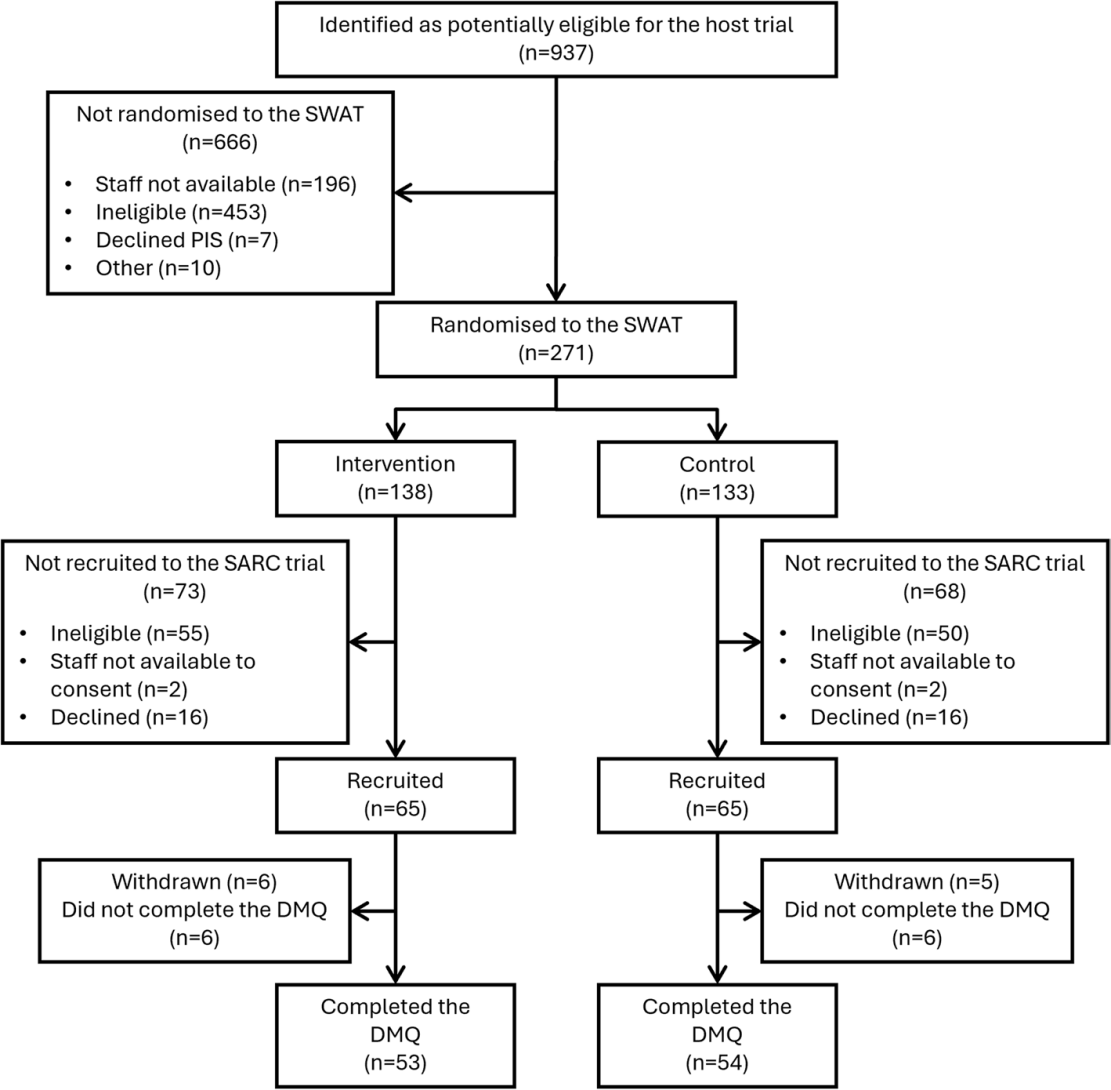
CONSORT Flow Diagram for SWAT participants

Many patients identified as potentially eligible for the host trial were not approached for consent, primarily due to subsequent ineligibility or limitations in staff availability (Fig. 1). A total of 271 participants were randomised to the SWAT, 138 of whom received the “optimised” PIL, and 133 received the conventional PIL. The DMQ was completed by 107 of the 130 participants who consented to take part in the host trial; reasons for non-completion of the DMQ were not captured.

We assessed adherence to the randomisation order and found that 19 envelopes (7%) were missed; of these, 7 were the optimised PIL and 12 were the conventional PIL. Reasons for errors in the application of the allocation sequences were not captured; key issues are suspected to be simple human error, the busy environment in which the trial was taking place, and the decision to use opaque envelopes for allocation concealment.

Participants had a median age of 44 (IQR 33–54) and age appeared similar between the two groups (optimised PIL median 41.5, IQR 33–52; conventional PIL median 46, IQR 33–56, *p* = 0.08).

### Interim analysis

The planned interim analysis took place after 50 participants had been randomised to the SWAT. Although recruitment was higher for the optimised PIL (18/24, 75%) than for conventional PIL (16/26, 61.5%), a chi-squared test indicated that this was not statistically significant (odds ratio (OR) 1.875; 95% CI 0.556 to 6.324, *p* = 0.308).

### Primary outcome analysis

Of the 271 participants recruited to the SWAT, 130 (48%) were recruited to SARC; of these, 65/138 (47%) received the optimised PIL, and 65/133 (49%) received the conventional PIL. Primary analysis using a chi-squared test yielded a *p*-value of 0.771 (Table 2).

**Table 2:** Primary analysis—chi-squared analysis

**Patient recruited?** *n* (%)	**Optimised PIL** (*n* = 138)	**Conventional PIL** (*n* = 133)	***p*-value**
Yes	65 (47%)	65 (49%)	0.771

A secondary analysis used logistic regression adjusted for age, yielding an odds ratio of 1.079 (95% CI 0.66 8 to 1.743, *p* = 0.755), suggesting that participants who received the optimised PIL were no more or less likely to be recruited to the SARC trial (Table 3).

**Table 3:** Secondary analysis—logistic regression including age as a covariate

**Patient recruited?** *N* = 271	**Odds ratio**	**95% CI**	***p*-value**
Conventional PIL	1.079	(0.668 to 1.743)	0.755
Age	0.998	(0.980 to 1.016)	0.836

### Secondary outcomes

A total of 107 participants completed the Decision-Making Questionnaire (DMQ), 53 in the optimised PIL group, 54 in the conventional PIL group. Four participants failed to complete all questions; the remainder returned a complete data set. Overall, the responses from participants were positive, with no clear differences between the two groups (Table 4).

**Table 4:** Comparison of the responses to the Decision-Making Questionnaire

		**Very hard**	**Hard**	**OK**	**Easy**	**Very easy**	**Total**
1. The information I saw about the *SARC* trial was easy to understand	Optimised	0 (0.0)	0 (0.0)	5 (9.4)	19 (36.0)	29 (54.7)	53
	Conventional	1 (1.9)	0 (0.0)	6 (11.1)	22 (40.7)	25 (46.3)	54
	Overall	1 (0.9)	0 (0.0)	11 (10.3)	41 (38.3)	54 (50.5)	107
	***p*-value**	**0.402**					
		**Not at all**	**Not really**	**Not sure**	**Yes mostly**	**Yes completely**	**Total**
2. After seeing the information about the *SARC* trial I knew what taking part would be like	Optimised	0 (0.0)	1 (1.9)	0 (0.0)	13 (24.5)	39 (73.6)	53
	Conventional	0 (0.0)	0 (0.0)	3 (5.6)	22 (40.7)	29 (53.7)	54
	Overall	0 (0.0)	0 (0.0)	3 (2.8)	35 (32.7)	68 (63.6)	107
	***p*-value**	**0.069**					
3. The information helped me understand how my treatment or care might change if I took part in the *SARC* trial	Optimised	0 (0.0)	0 (0.0)	1 (1.9)	19 (35.8)	33 (62.3)	53
	Conventional	0 (0.0)	0 (0.0)	3 (5.6)	12 (22.2)	39 (72.2)	54
	Overall	0 (0.0)	0 (0.0)	4 (3.7)	31 (29.0)	72 (67.3)	107
	***p*-value**	**0.455**					
4. The possible benefits of taking part in the *SARC* trial were made clear in the information	Optimised	0 (0.0)	0 (0.0)	0 (0.0)	7 (13.2)	46 (86.8)	53
	Conventional	0 (0.0)	0 (0.0)	0 (0.0)	9 (16.7)	45 (83.3)	54
	Overall	0 (0.0)	0 (0.0)	0 (0.0)	16 (15.0)	91 (85.0)	107
	***p*-value**	**0.787**					
5. The possible disadvantages of taking part in the *SARC* trial were made clear in the information	Optimised	0 (0.0)	1 (1.9)	1 (1.9)	12 (22.6)	39 (73.6)	53
	Conventional	0 (0.0)	0 (0.0)	0 (0.0)	16 (29.6)	38 (70.4)	54
	Overall	0 (0.0)	1 (0.9)	1 (0.9)	28 (26.2)	77 (72.0)	107
	***p*-value**	**0.852**					
6. The information about the *SARC* trial helped me discuss the trial with the person who asked me to take part (usually a doctor, nurse or researcher)	Optimised	0 (0.0)	0 (0.0)	0 (0.0)	7 (13.2)	46 (86.8)	53
	Conventional	0 (0.0)	0 (0.0)	1 (1.9)	10 (18.5)	43 (79.6)	54
	Overall	0 (0.0)	0 (0.0)	1 (0.9)	17 (15.9)	89 (83.2)	107
	***p*-value**	**0.509**					
7. I am confident that I have made the right decision about whether or not to take part in the *SARC* trial	Optimised	0 (0.0)	0 (0.0)	0 (0.0)	7 (13.7)	44 (86.3)	51
	Conventional	0 (0.0)	0 (0.0)	0 (0.0)	8 (15.1)	45 (84.9)	53
	Overall	0 (0.0)	0 (0.0)	0 (0.0)	15 (14.4)	89 (85.6)	104
	***p*-value**	**0.532**					
8. In all, the information about the *SARC* trial helped me make my decision about whether or not to take part	Optimised	0 (0.0)	0 (0.0)	1 (2.0)	6 (11.8)	44 (86.3)	51
	Conventional	0 (0.0)	0 (0.0)	1 (1.9)	15 (28.3)	37 (69.8)	53
	Overall	0 (0.0)	0 (0.0)	2 (1.9)	21 (20.2)	81 (77.9)	104
	***p*-value**	**0.156**					
		**No**		**Yes**		**Total**	
Was there anything you wanted to know about the *SARC* trial but which was not included in the information you saw?	Optimised	45 (90.0)		5 (10.0)		50	
	Conventional	50 (94.3)		3 (5.7)		53	
	Overall	95 (92.2)		8 (7.8)		103	
	***p*-value**	**0.480**					

## Discussion

The optimised PIL given to patients did not impact on the host trial’s recruitment rate in this SWAT. Analysis of the decision-making questionnaire responses suggested that improving the readability and presentation of the PIL did not impact participants’ decision-making about trial participation. These results are consistent with previous research that indicated modifying the design of the PIL did not improve recruitment [14].

Whilst improvements to length, readability and presentation of study information may not improve recruitment, we must remember the ethical imperative to ensure participants provide truly informed consent for research. This will require different approaches depending on the complexity of the study, the burden on participants, the eligible patient cohort, the context in which they are recruited, and the risks and potential benefits associated with participation. This SWAT was conducted before the HRA released their Patient Information Design and Review Principles [12]; many of the changes implemented in the optimised PIS are also suggested in this document (e.g. improve readability using the Flesch-Kincaid grade level). The optimised PIL had an improved readability score but was still above the reading age of nearly half of adults in the UK, which is the equivalent to that of an 11–14-year-old [8]. Whilst both PILs were reviewed by the PPI group for the host trial, a structured, co-designed approach may have further improved the readability and design, and although previous research suggests this would not impact recruitment [14], it may have affected the results of the Decision-Making Questionnaire. This may be an opportunity for further research into this area, in an effort to continue to improve the accessibility of information in a more empirical fashion. Changes to the standard PIL were hampered by Sponsor and regulatory requirements, as the host trial was a Clinical Trial of an Investigational Medicinal Product (CTIMP). At the time, the GDPR transparency wording required by the HRA was lengthy and any potential for modification was limited.

## Conclusion

Optimising the PIL for readability had no impact on recruitment to an interventional trial set in an Emergency Department, nor on the decision-making of potential participants. Whilst improving the readability and presentation of information provided to potential participants of clinical studies should remain a priority for researchers and funders, it appears to be an ineffective strategy for improving recruitment rates.

## Limitations

This was a single-site, single-host trial SWAT, and therefore has limited generalisability. The SWAT methodology was labour-intensive and would not be well-suited to multi-centre studies.

The Decision-Making Questionnaire was provided to participants after they had consented to take part in the host trial; the views of approached participants who declined to take part in the host trial were therefore not captured.

## Registration

The SWAT is registered as SWAT101 on the Northern Ireland Methodology Hub’s SWAT repository, registered December 2018, https://www.qub.ac.uk/sites/TheNorthernIrelandNetworkforTrialsMethodologyResearch/FileStore/Filetoupload,926067,en.pdf. The host trial is registered on the ISRCTN trial registry, number ISRCTN14552440. The protocol for the host trial, which includes the protocol for the SWAT, can be found on the ISRCTN record.

## Supplementary Information

Additional file 1: Optimised PIL (PIL A) and Conventional PIL (PIL B).

Additional file 2: Decision Making Questionnaire.

## Data Availability

The datasets generated and/or analysed during the current study and be available upon request from the Sponsor, uhdb.sponsor@nhs.net.
