# Supplementary material for: A randomised Study Within a Trial (SWAT) to determine if participant information leaflet design affects recruitment rate into an interventional trial taking place in a UK emergency department
Source: Trials. 2026 Feb 6;27:201. doi: 10.1186/s13063-025-09412-6 (PMC12977681; doi:10.1186/s13063-025-09412-6)
Supplement: Supplementary file 1 — Additional file 1: Optimised PIL (PIL A) and Conventional PIL (PIL B). [file 13063_2025_9412_MOESM1_ESM.pdf]

6) What will happen with the information collected about me?

Any information you give us will be kept strictly confidential. The team at University Hospitals of Derby & Burton NHS Foundation Trust (UHDB) will work according to the Data Protection Act 2018 and the General Data Protection Regulations (GDPR).

- UHDB is the sponsor for this trial and will act as the data controller. This means that we are responsible for looking after your information and using it properly.
- The research team will collect information from you and your medical records for this trial in accordance with instructions from us.
- They will use your **name, hospital number and contact details** to contact you about the trial make sure that relevant information is recorded.
- UHDB will keep identifiable information about you from this trial for **15 years** after the trial has finished. To safeguard your rights, we will use the minimum personally-identifiable information possible.
- **Your rights to access, change or move your information are limited**, as we need to manage your information in specific ways in order for the research to be reliable and accurate.
- **If you withdraw** from the trial, we will keep the information about you that we have already obtained and it will not be possible to identify you from this.
- Individuals from UHDB and regulatory organisations may look at your medical records and the information collected about you for this trial. This is to help them check the data collection process and ensure the trial is being carried out as it should be.
- The people who analyse the information will not be able to identify you and will not be able to find out your name, hospital number or contact details.

You can find out more about how we use your information by visiting:

https://www.uhdb.nhs.uk/research-how-we-use-your-information.

7) What do I have to do now?

If you have no other questions and are happy to take part in the trial, then you will be asked to sign a consent form.

8) Contacts for more information

If you need any more information, please contact us.

Please contact one of the contacts below or feel free to discuss this study with any health care professional involved in you care.

Contact details

Chief Investigator: Dr Graham Johnson

Telephone:

Insert Research Team Contact Details:

REMEDY:

Thank you for reading this information.
If you decide to take part, please keep this leaflet.

participant information leaflet

SARC

Salbutamol for Analgesia in Renal Colic: A prospective, randomised, placebo controlled Phase II trial

We would like to invite you to take part in a clinical trial

Important things you need to know

- This trial is looking at a new way of managing the pain of renal colic (kidney stones)
- People attending A&E with pain that we believe to be caused by renal colic are being asked if they would like to take part in this trial.
- Those who take part will receive routine care as well as an injection of either salbutamol or placebo; which injection they receive is randomly decided.
- We are then asking patients to answer some questions based on their pain levels to help us judge whether salbutamol had any impact on pain relief.
- Participation is completely voluntary and you can change your mind at any time.

Contents

1) Why are we doing this study?
2) What will happen if I take part?
3) Possible advantages and disadvantages of taking part
4) About salbutamol
5) More information about taking part
6) What will happen to the information collected about me?
7) What do I have to do now?
8) Contacts for more information

Before you decide...

- Please take time to read the following information carefully and discuss it with others if you wish
- Ask us if you are unsure of anything you read in this information leaflet. Our contact details are on the back of this leaflet.
- You are free to decide whether or not to take part. If you choose not to take part, this will not affect any care you may get.

Please turn over to read the full information about the trial

SARC Participant Information Leaflet v2.0 15.JUL.2019
IRAS ID: 252075

NHS
University Hospitals of Derby and Burton
NHS Foundation Trust

1) Why are we doing this trial?

Renal colic is the name for the pain experienced when a kidney stone causes a blockage in the tube between the kidney and the bladder.

Previous groups of patients who have experienced renal colic have told us how painful renal colic can be, how long the pain-killers take to be effective, and how unpleasant the side effects can be.

We think that salbutamol, a drug commonly and successfully used to treat asthma, may reduce the pain of renal colic. It has few side effects.

This trial will investigate whether adding salbutamol to the normal pain relief given to patients with renal colic leads to better pain control .

Why am I being asked to take part?

Your doctor believes that you are experiencing renal colic, pain caused by kidney stones, so this makes you potentially eligible to take part in this trial. At the Royal Derby Hospital we treat around 400 patients a year with renal colic. We are hoping that 118 of them will take part in with this trial.

What will happen in this trial?

To discover whether salbutamol can be effective for pain relief, we will randomly put patients in to one of two groups, either to receive salbutamol or placebo (sodium chloride—salty water that's safe for injection). This is done by chance, like the toss of a coin.

2) What will happen if I take part?

If you would like to take part, we will make sure that you have understood this information and asked any questions you may have, and will then ask you to sign a consent form.

- The usual care you would receive as someone with suspected renal colic will continue.
- You will be given the usual pain relief you would expect to receive (you may have already received some of this pain relief).
- You will have an electrocardiogram (ECG—a heart tracing) if you have not already had one. We may also need to take an additional blood test to confirm you are eligible for the study (we won't keep the blood sample).
- We will ask you to complete a couple of questionnaires about your pain, called the McGill questionnaire and a Visual Analogue Scale (VAS).
- The clinical staff looking after you will to take a note of your heart rate, breathing rate, and blood pressure, as well as other observations you would normally expect to have taken.
- You will then receive **one** injection over 3-5 minutes, given by hand, of either salbutamol or placebo (salty water).
- You will have a 50:50 chance of receiving salbutamol, and neither you nor the team looking after you will know which treatment you receive.
- The trial will take place over a 24 hour period. During this time you will have regular observations taken and will be asked to answer questions on how bad you pain is multiple times over this period.
- If you are ready for discharge sooner than 24 hours then the study will stop at this stage.

3) Possible advantages and disadvantages of taking part

We cannot promise that taking part in this trial will help you. We think that salbutamol will help with pain relief, but we don't know for sure, and this is why we are doing this trial. We hope that the results of this trial will help us to go on to do a much larger trial to gather more data on this.

Taking part will involve some of your time to fill in the questionnaires about your pain, but you will not be kept in hospital longer than would normally be necessary due to taking part.

More information on salbutamol is in the next section.

4) About salbutamol

Salbutamol is a well-understood and safe medicine, but it still has some side effects. If it is unsafe for you to take part in this trial or salbutamol would interact with any of your normal medicines then you will not be asked to take part.

A number of people who are given salbutamol will be shaky, have a fast heart beat, develop a headache or have muscle cramps. These side effects are usually mild and are always short lived.

If you are, or think you might be pregnant, are breast feeding or trying to get pregnant, please inform the research team as it may not be appropriate for you to take part.

If you are asthmatic and have taken your inhaler in the last 6 hours, please let a member of trial staff know.

5) More information about the trial

Do I have to take part?

No—it is entirely up to you whether or not you take part in this trial. Saying no will not impact on the usual care you would expect to receive.

What if I change my mind?

Even if you decide to take part, but change your mind either before or after you've received the injection, we will stop collecting information from you. You don't have to give a reason.

What if there is a problem?

If you are worried or have any questions about any part of this trial, you can call the lead investigator, Dr Graham Johnson.

If you want to speak to someone independent, or if you have any concerns or a complaint, you should contact:

- Your local patient advice and liaison service (PALS) on xxxxx

The normal NHS complaints mechanisms are available to you.

We don't expect it to, but in the event that something does go wrong and you are harmed because of the fault of someone, then you may have grounds for legal action for compensation against UHDB, but you may have to pay your legal costs.

Will my GP be involved?

If you agree, we will let your GP know that you are taking part in this trial. There is a box on the consent form for you to sign to agree to this.

Who is organising and funding this trial?

This trial is organised by Derby Clinical Trials Support Unit on behalf of University Hospitals of Derby and Burton NHS Foundation Trust (UHDB) and funded by the National Institute for Health Research (NIHR).

All research in the NHS is looked at by a number of different bodies before it can start, the purpose of which is to ensure that the trial meets ethical and legal standards and the safety, rights and well-being of participants are protected.

This trial has been reviewed and approved by:

- West of Scotland Research Ethics Committee (REC) 1, an independent group or experts and lay members.
- The Health Research Authority (HRA).
- The Medicines and Healthcare products Regulatory Authority (MHRA).

Please turn over

Salbutamol for Analgesia in Renal Colic: A prospective, randomised, placebo controlled Phase II trial

IRAS ID: 252075

Participant Information Leaflet

SARC Study

We would like to invite you to take part in a research study. Before you decide whether or not to take part, your medical team will go through this patient information leaflet with you. They will answer any questions you may have so that you fully understand why we are running the study and what it would involve for you.

Please take the time to read the information carefully and talk to others about the study if you wish. Ask your doctor or nurse if there is anything you don’t understand or if you would like more information. Please take your time to decide whether or not you wish to take part.

You are free to decide if you want to take part in this research study. If you choose not to take part this will not affect the usual care you receive in any way.

You can decide to stop taking part in the study at any time without giving a reason. If you decide to take part, we will ask you to sign a form to give your consent for the study.

Contents

1. What is the purpose of the study
2. Why have I been invited?
3. Do I have to take part?
4. What will happen to me if I take part?
5. What will I have to do?
6. Expenses
7. What are the disadvantages and risks of taking part?
8. What are the advantages of taking part?
9. What happens when the study stops?
10. What if there is a problem?
11. Will my participation in the study be kept confidential?
12. What will happen to the results of the research study?
13. Who has reviewed this research?
14. Who is organising and funding the research?
15. Contacts for further information

1. What is the purpose of the study?

Renal colic is the name for the pain experienced when a kidney stone causes a blockage in the tube between the kidney and the bladder.

Standard pain-killers do not always work and are also associated with side effects such as vomiting or drowsiness. Previous groups of patients who have experienced renal colic have described how bad the pain from renal colic can be, how long the pain-killers take to be effective, and how unpleasant the side effects can be.

It is thought that salbutamol, a drug commonly and successfully used to treat asthma, may reduce the pain of renal colic. It has few side effects.

This study will investigate whether adding salbutamol to the normal pain relief given to patients with renal colic leads to better pain control.

2. Why have I been invited?

You have been invited because you have pain in your abdomen or flank and your medical team suspect that you may have renal colic.

3. Do I have to take part?

No. It is up to you to decide whether to take part. Even if you decide to take part, but change your mind at any time, you will be free to withdraw at any time and without giving a reason. Your usual care will not be affected in any way whether or not you take part, or even if you take part and then decide to withdraw. If you do withdraw from the study, we will keep any information relating to the study which has been collected about you up to that point. This information will, however, be anonymised so that you will not be identifiable from it in any way.

If you do decide to take part, we will ensure that you understand all the information provided, and you will then be asked to sign a consent form. You will be given a copy of the signed consent form to keep, as well as a copy of this information leaflet.

4. What will happen to me if I take part?

Routine assessments to determine whether you are experiencing renal colic will continue and you will be given the usual pain relief as part of normal medical care. We may need to do an ECG (a heart tracing) and a blood test to ensure you are eligible to take part; this will happen only if you haven't already had these done and you wish to take part in the trial. We will not keep the blood sample we take once it has been processed.

If you are found to be eligible to take part then a doctor will discuss the study with you. During this time you will have the opportunity to ask any questions or discuss worries you may have. After this, should you wish to take part, you will be asked to sign a form to say that you consent to be part of the study. Once you have agreed to take part, we will make arrangements for you to start the study medication.

In this study one group of patients will receive usual standard care for their pain (according to the best way we know how at the moment) and placebo (salty water that's safe for injection) and the other group will receive the same standard care and intravenous salbutamol (into a drip). This lets us compare the results between the two groups to see which is better. To try and make sure the groups are the same to

start with, each patient is put into a group by chance (randomly, like the toss of a coin). You will have a 50:50 chance of being allocated to the salbutamol group, and to minimise the chance of bias neither you nor the team looking after you will know which treatment you receive.

The study will take place over a 24 hour period. During this time you will have regular observations taken at the time the trial drug is given and then at 15 and 30minutes, 1, 2, 4, 8, 12, 16, 20 and 24 hours. If you are ready to go home from hospital sooner than 24 hours then the study will finish at this time; you will not be kept in hospital longer than would normally be necessary due to participation in this study.

5. What will I have to do?

You will be asked to answer questions about your pain at the time points discussed above. You will also be asked to complete a questionnaire about your involvement in the study.

6. Expenses

Taking part will not cost you anything, and no payments will be made as a result of this trial.

7. What are the disadvantages and risks of taking part?

The risk of taking part in the trial is no higher than the risk of standard medical care.

Salbutamol is a commonly used drug with few significant side effects. A number of people who are given salbutamol will be shaky, have a fast heart-beat, develop a headache or have muscle cramps. These side effects are usually mild, and are always short-lived.

If you are pregnant, breast-feeding or trying to get pregnant then please inform the research staff as it may not be appropriate for you to take part in the trial.

If you are asthmatic and have taken your inhaler in the last 6 hours, please let a member of trial staff know.

8. What are the advantages of taking part?

We cannot be certain that there will be any advantages to you being in the trial, although salbutamol may reduce the pain of renal colic. We hope that the information gained from this trial will help future patients with renal colic.

9. What happens when the trial stops?

The routine clinical care of both you and other people will not be affected in any way. Your pain will be investigated and any necessary follow-up arranged exactly as if you had not taken part in the trial.

10. What if there is a problem?

If you have any concerns or queries about any aspect of this study you should ask to speak to one of the research team or your Doctor who will be undertaking the day-to-day running of the study and who will do their best to answer your questions.

We don't expect it to, but in the event that something does go wrong and you are harmed during the research and this is due to someone's negligence, then you may have grounds for legal action against University Hospitals of Derby and Burton NHS Foundation Trust, but you may have to pay your legal costs. The normal National Health Service complaints mechanisms will still be available to you (if appropriate).

If you wish to complain about the conduct of the research you should contact Dr Graham Johnson, Department of Emergency Medicine, Royal Derby Hospitals, University Hospitals of Derby and Burton NHS Foundation Trust, Derby, DE22 3NE. Telephone 01332 340131

Alternatively, if you wish to speak with someone not involved in the study, you could contact the Patient Advice and Liaison Service (PALS) on:

Freephone:
Email:
Text:

11. Will my participation in the study be kept confidential?

Yes. We will follow all relevant ethical and legal practices and all information collected about you during the course of the research will be handled confidentially. It is necessary to record in your hospital notes that you are participating in this study, for your benefit and protection. The team at University Hospitals of Derby & Burton NHS Foundation Trust (UHDB) will work according to the Data Protection Act 2018 and the General Data Protection Regulations (GDPR).

UHDB is the sponsor for this trial and will act as the data controller. This means that we are responsible for looking after your information and using it properly.

The research team will collect information from you and your medical records for this trial in accordance with instructions from us. They will use your name, hospital number and contact details to contact you about the trial make sure that relevant information is recorded. You will be allocated a study number, and this along with your initials will be the only references that will be recorded about you on our computers.

UHDB will keep identifiable information about you from this trial for 15 years after the trial has finished. To safeguard your rights, we will use the minimum personally-identifiable information possible. Your rights to access, change or move your information are limited, as we need to manage your information in specific ways in order for the research to be reliable and accurate.

Information about your participation in this study will be sent to your GP.

If you withdraw from the trial, we will keep the information about you that we have already obtained and it will not be possible to identify you from this.

Individuals from UHDB and regulatory organisations may look at your medical records and the information collected about you for this trial. This is to help them check the data collection process and ensure the trial is being carried out as it should be. The people who analyse the information will not be able to identify you and will not be able to find out your name, hospital number or contact details.

12. What will happen to the results of the research study?

You can choose to withdraw from the study at any time you wish and without having to give us a reason. If you do, we will need to use the data collected on you up to that point but this will of course be kept confidential and you cannot be identified

from it.. If you choose to withdraw from the study this will not affect your on-going routine clinical care and we will make arrangements for this to be restarted for you.

Once the study is complete its results will be analysed and published in a medical journal, as well as presented at conferences. You will not be identifiable in any of these presentations or publications.

13.Who has reviewed this research?

All research in the NHS is looked at by independent group of people called a Research Ethics Committee, to protect your interests. This study has been reviewed and given favourable opinion by the West of Scotland Research Ethics Committee 1.

14. Who is organising and funding the research?

The study has been designed by a group led by Dr Graham Johnson, Consultant in Emergency Medicine at University Hospitals of Derby and Burton NHS Foundation Trust. It is funded by a grant from the National Institute for Health Research's Research for Patient Benefit Programme.

15. Contacts for further information

XXXXXX
XXX
XX
XXX
XXX

Insert Research Team Contact details
Address:
Telephone Number:

Please contact one of the contacts above or feel free to discuss this study with any health care professional involved in your care.

If you wish to discuss the study with someone independent of the research team you can contact the local NHS Patient Advice and Liaison Service (PALS) on:

Email: Phone:

To find out more about the regulation of Research within the NHS visit:
www.nres.nhs.uk
