# Supplementary material for: A randomised Study Within a Trial (SWAT) to determine if participant information leaflet design affects recruitment rate into an interventional trial taking place in a UK emergency department
Source: Trials. 2026 Feb 6;27:201. doi: 10.1186/s13063-025-09412-6 (PMC12977681; doi:10.1186/s13063-025-09412-6)
Supplement: Supplementary file 2 — Additional file 2: Decision Making Questionnaire. [file 13063_2025_9412_MOESM2_ESM.pdf]

DECISION-MAKING QUESTIONNAIRE
We would like to know your views on the information you were given about *SARC* and how much it helped your decision about taking part in the trial.<br>Completion of this questionnaire is completely voluntary and your treatment will not be affected in any way by your decision whether or not to complete it.

**Participant ID:**																
**Date:**			/				/					**Time (24h)**			:		

- Below, there are 8 statements about the information that you were given for SARC.
- For each one, please put a circle around the option that best matches your view.
- In other words, show us how much you may agree or disagree with a statement.

The information I saw about the *SARC* trial was easy to understand.

Very Hard   Hard   OK   Easy   Very easy

After seeing the information about the *SARC* trial I knew what taking part would be like.

Not at all   Not really   Not sure   Yes mostly   Yes completely

The information helped me understand how my treatment or care might change if I took part in the *SARC* trial.

Not at all   Not really   Not sure   Yes mostly   Yes completely

The possible benefits of taking part in the *SARC* trial were made clear in the information.

Not at all   Not really   Not sure   Yes mostly   Yes completely

The possible disadvantages of taking part in the *SARC* trial were made clear in the information.

Not at all   Not really   Not sure   Yes mostly   Yes completely

The information about the *SARC* trial helped me discuss the trial with the person who asked me to take part (usually a doctor, nurse or researcher).

Not at all   Not really   Not sure   Yes mostly   Yes completely

PLEASE TURN OVER

I am confident that I have made the right decision about whether or not to take part in the *SARC* trial.

Not at all Not really Not sure Yes mostly Yes completely

In all, the information about the *SARC* trial helped me make my decision about whether or not to take part.

Not at all Not really Not sure Yes mostly Yes completely

Was there anything you wanted to know about the *SARC* trial but which wasn't included in the information you saw?

Yes / No

If yes, please write them here:

____

____

____

____

Thank you for taking the time to complete this questionnaire.
